# Germline *TERT* promoter mutations are rare in familial melanoma

**DOI:** 10.1007/s10689-015-9841-9

**Published:** 2015-10-03

**Authors:** Mark Harland, Mia Petljak, Carla Daniela Robles-Espinoza, Zhihao Ding, Nelleke A. Gruis, Remco van Doorn, Karen A. Pooley, Alison M. Dunning, Lauren G. Aoude, Karin A. W. Wadt, Anne-Marie Gerdes, Kevin M. Brown, Nicholas K. Hayward, Julia A. Newton-Bishop, David J. Adams, D. Timothy Bishop

**Affiliations:** Section of Epidemiology and Biostatistics, Leeds Institute of Cancer and Pathology, University of Leeds, Leeds, LS9 7TF UK; Experimental Cancer Genetics, The Wellcome Trust Sanger Institute, Hinxton, Cambridge, CB10 1SA UK; Department of Dermatology, Leiden University Medical Center, Leiden, The Netherlands; Strangeways Research Laboratory, Departments of Public Health and Primary Care, Centre for Cancer Genetic Epidemiology, University of Cambridge, 2, Worts Causeway, Cambridge, CB1 8RN UK; Strangeways Research Laboratory, Departments of Oncology, Centre for Cancer Genetic Epidemiology, University of Cambridge, 2, Worts Causeway, Cambridge, CB1 8RN UK; Oncogenomics Laboratory, QIMR Berghofer Medical Research Institute, Herston, Brisbane, QLD Australia; Department of Clinical Genetics, University Hospital of Copenhagen, Copenhagen, Denmark; Laboratory of Translational Genomics, Division of Cancer Epidemiology and Genetics, National Cancer Institute, Bethesda, MD USA

**Keywords:** Melanoma, Familial, Genetic, TERT, Mutation

## Abstract

Germline *CDKN2A* mutations occur in 40 % of 3-or-more case melanoma families while mutations of *CDK4*, *BAP1*, and genes involved in telomere function (*ACD, TERF2IP,POT1*), have also been implicated in melanomagenesis. Mutation of the promoter of the telomerase reverse transcriptase (*TERT*) gene (c.−57 T>G variant) has been reported in one family. We tested for the *TERT* promoter variant in 675 multicase families wild-type for the known high penetrance familial melanoma genes, 1863 UK population-based melanoma cases and 529 controls. Germline lymphocyte telomere length was estimated in carriers. The c.−57 T>G *TERT* promoter variant was identified in one 7-case family with multiple primaries and early age of onset (earliest, 15 years) but not among population cases or controls. One family member had multiple primary melanomas, basal cell carcinomas and a bladder tumour. The blood leukocyte telomere length of a carrier was similar to wild-type cases. We provide evidence confirming that a rare promoter variant of *TERT* (c.−57 T>G) is associated with high penetrance, early onset melanoma and potentially other cancers, and explains <1 % of UK melanoma multicase families. The identification of *POT1* and *TERT* germline mutations highlights the importance of telomere integrity in melanoma biology.

**Electronic supplementary material:**

The online version of this article (doi:10.1007/s10689-015-9841-9) contains supplementary material, which is available to authorized users.

## Background

Cutaneous melanoma occurs predominantly in the genetically predisposed, i.e. white-skinned individuals with skin that burns easily with UV exposure, and who are prone to develop melanocytic naevi [2, 3]. Genome-wide association studies confirm that the strongest melanoma-associated single nucleotide polymorphisms (SNPs) align with these melanoma-associated risk phenotypes [4].

Clustering of melanoma in families however points to rare high-penetrance dominantly inherited mutations. The most frequently mutated high-penetrance melanoma susceptibility gene is *CDKN2A* (cyclin-dependent kinase inhibitor 2A), which encodes tumour suppressors p16 and p14ARF. Worldwide*, CDKN2A* mutations explain predisposition in approximately 40 % of families with three or more cases of melanoma, ranging from 20 % in Australia to 57 % in Europe [5]. The proportion of families with identifiable *CDKN2A* mutations varies geographically, being lower in countries with high ambient solar ultraviolet radiation populated by fair-skinned people [5]. *CDKN2A* mutation carriers tend to have multiple primary melanomas, early onset melanoma and an increased risk of developing pancreatic cancer [5] and have also been shown to have an increased risk of tobacco associated cancers in respiratory and digestive tissues [6, 7]. A very small proportion (<1 %) of families worldwide have been reported with mutations in the *CDK4* (Cyclin-dependent kinase 4) gene at a key p16-binding residue [8].

Since germline *CDKN2A* mutations were first identified in families there has been considerable effort to identify additional causal (*i.e.* high-penetrance) mutations in novel genes. The Melanoma Genetics Consortium (GenoMEL, www.genomel.org) has identified a number of genes in which mutations appear to predispose to melanoma, however the number of families in which these novel genes are involved is few. Rare families susceptible to uveal and cutaneous melanoma and non-melanoma cancers such as mesothelioma and renal cell carcinoma have also been described with inactivation of BRCA1 associated protein 1 (*BAP1*) [9], a gene also frequently somatically mutated in uveal melanomas [10]. More recently, rare germline mutations in the protection of telomeres 1 gene (*POT1*) were found in families with early onset and multiple primary melanomas [11, 12]. The reported mutations abolish the binding of POT1 (part of the shelterin complex) to telomeres, leading to fragile telomeres and increased telomere length, a reported association with melanoma predisposition [13]. Mutations have also been found in other members of the shelterin complex (*ACD* and *TERF2IP*) in melanoma families [14]. Despite this progress, however, a high proportion (around 60 %) of families worldwide cannot be explained by predisposing mutations in known genes.

Horn et al. [1] recently identified a germline mutation in the promoter of *TERT*, −57 bp (chr5:1295161 T>G, GRCh37/hg19) from the translation start site (hereinafter referred to as c.−57 T>G) co-segregating with melanoma in a 14-case family. This family was characterized by early age of onset (average 34 years) of melanoma and evidence of susceptibility to other cancers. Two melanoma cases developed ovarian cancer and a third had 5 different primary cancers other than melanoma (ovary, renal cell carcinoma, bladder, breast and lung). Further, somatic mutations within the *TERT* promoter, that (similarly to the familial c.−57 T>G variant) create a new ETS transcription factor binding site, occur in a high proportion of melanomas, supporting the important role of telomeres and *TERT* in melanoma [15].

The inheritance of common genetic variation that predicts telomere length is a risk factor for melanoma [16] and the identification of telomere-related genes (*POT1*, *TERT, ACD and TERF2IP*) as highly penetrant susceptibility genes in melanoma families suggests that telomere regulation has considerable biological relevance in melanoma. *POT1* germline carriers have longer germline telomeres than matched controls [11] there is as yet no published evidence with regard to telomere length for individuals carrying *TERT* promoter variants.

## Methods

### *TERT* promoter analyses

#### Screening for mutations in melanoma families

Melanoma families were recruited in Leeds, UK (data protection and Ethical Committee reference NIGB MR64, MREC 99/3/45), Copenhagen, Denmark (Ethics Committee of the capitol region of Copenhagen, H-3-2011-050), Brisbane, Australia (approved by QIMR Berghofer Human Research Ethics Committee) and Leiden, Netherlands (ethics approval number, P00.117).

Germline DNA from the probands of 67 UK, 16 Danish, 169 Australian, and 21 Dutch families (total of 273 families) with 3 or more cases of melanoma without known high penetrance mutations (*CDKN2A, CDK4, BAP1, POT1, ACD, TERF2IP*) were screened. A further 402 families consisting of 2 cases of melanoma recruited in the UK (93 families), Denmark (72 families) and Australia (237 families) were also screened.

The *TERT* promoter critical region (encompassing −57 to −149 bp) was amplified and capillary-sequenced using the same primers as described in Horn et al. [1], to examine the c.−57 T>G germline mutation and the two most prevalent somatic mutations (c.−124 G>A and c.−146 G>A from the translation start site [TSS]; these mutations were termed “228” and “250” in [1] reflecting their absolute basepair location rather than as here to the TSS).

#### Screening samples from population-based cases and controls

In order to estimate the prevalence of germline *TERT* promoter mutations in population-ascertained melanoma cases, we screened blood-derived DNA from 1863 cases and 529 controls recruited to the Leeds Melanoma Case–Control study [17]. A custom Taqman® assay (Thermo Fisher Scientific; AHGJ4ZR) was used to screen these samples for the c.−57T>G *TERT* promoter variant. We also attempted to screen the 2 main sites somatically mutated using Taqman® assays but the assay for c.−146G>A failed manufacture (custom assay AHHS25Z for c.−124G>A).

#### Haplotype/relatedness studies

The 1.06 Mb risk haplotype around the c.−57 T>G *TERT* promoter mutation contains 6 rare SNP variants in the reported family [1]. To test if the variant is recurrent or a founder mutation, we examined the segregating haplotype for these 6 SNPs, and we conducted relatedness analysis based on the Illumina HumanCytoSNP-12v2_H array, publically available SNP allele frequencies and identity-by-descent analysis using PLINK [18].

### Telomere length assays

Telomere length (TL) data was available from two approaches: (A) from whole exome sequence data with a method described by Ding et al. [19] and (B) using a modified q-PCR approach. For (A) germline DNA samples from melanoma cases with a family history were subject to targeted pulldown prior to next generation sequencing. The algorithm estimates telomere length by counting the off-target telomeric repeat units and normalizing by the total number of reads with GC composition comparable to that of the telomere region (~50 %). TL was estimated for all 41 familial melanoma cases for whom whole exome sequencing was conducted at the Wellcome Trust Sanger Institute; this set included samples shown to have *POT1* germline mutations and previously reported showing that persons with germline *POT1* mutations associated with dominantly inherited susceptibility to melanoma had increased telomere length with these assays compared to cases without *POT1* mutations [11]. For (B), relative mean TL was also estimated by a SYBR^®^ Green real-time PCR using a version of the published q-PCR protocols modified as described previously comparing population-based cases with these families [20].

## Results

### c.−57 T>G *TERT* promoter variant

**Fig. 1:**
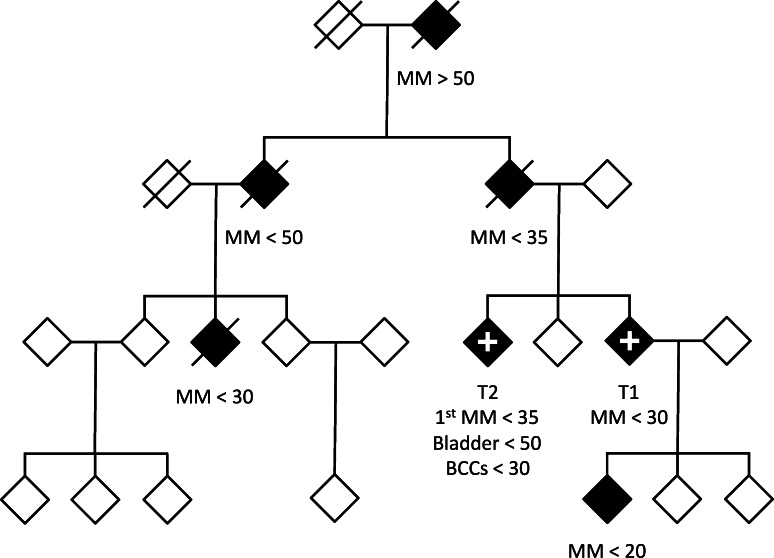
Pedigree of UK family with 7 cases of melanoma in which the c.−57 T>G *TERT* promoter variant was reported in 2 affected cases. Both tested samples carried the variant allele. *MM* shows diagnosis of first malignant melanoma. <n (>n) indicates first diagnosis before (after) age n years. *BCC* indicates basal cell carcinoma. *+ symbol* indicates germline sample carried c.−57 T>G variant. Other samples were not available for testing

The c.−57 T>G *TERT* promoter variant was identified in the proband of a single 7-case UK family and subsequently in a sibling (Fig. 1). The family was unusual within the UK in that the youngest case (not tested) was 15 years old at diagnosis, and one case had developed 7 basal cell carcinomas (an exceptionally large number for the UK) in addition to melanomas and early onset borderline bladder cancer. No case or control within the Leeds Melanoma case–control study carried the c.−57 T>G variant.

### Other promoter variants

The quality of the sequencing did not allow detailed examination of the whole of the region but we could assess the positions −57, −124 and −146 from the TSS confidently. No other variants were identified at these positions. Furthermore in the case control study there was no variation shown at position −124, a site commonly mutated somatically.

### Relatedness to family reported by Horn et al

Capillary sequencing of the UK family members showed that the variant-bearing haplotype did not contain any of the 6 rare variants seen in the reported family [1]. However, the two haplotypes do share the minor allele for rs2853669 (estimated allele frequency = 0.3), which lies in the promoter 188 bp upstream of the c.−57 T>G variant. To assess the significance, we examined the relatedness of the families. There was strong evidence against close relatedness of the UK family (at least 7th degree, data not shown) with the previously reported family, suggesting these are independently occurring mutations.

### Telomere length

**Fig. 2:**
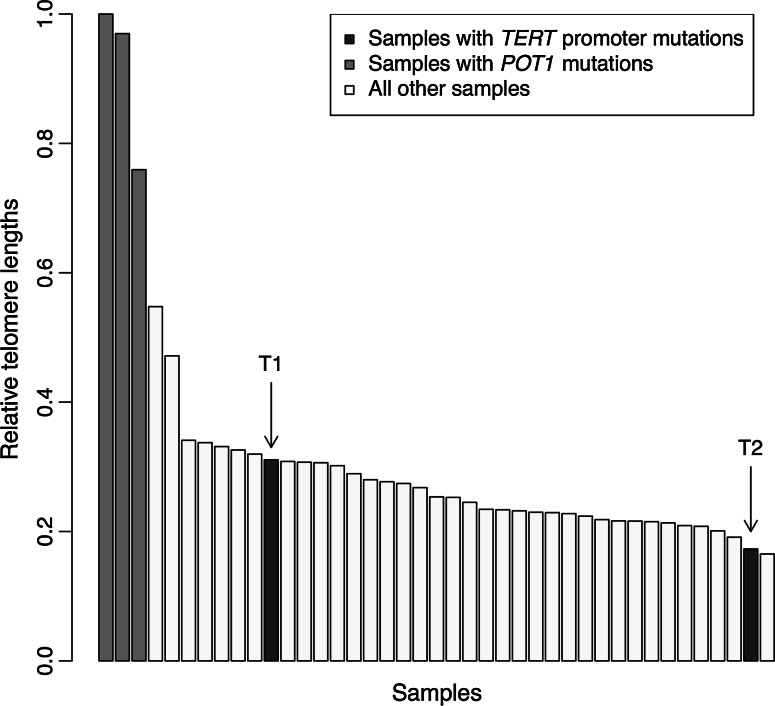
Estimated telomere length of familial melanoma cases relative to the telomere length of the case with the longest telomere. Adjustment for age and sex makes minimal difference to these results because of the limited age range of those recruited so these are the raw estimates from bioinformatic analysis of whole exome resequencing data [19]. Each sample reflects the germline DNA from a melanoma case also with a family history. T1 and T2 refer to the 2 tested samples in Fig. 1 with the *TERT* promoter variant. This figure is modified from Robles-Espinoza et al. [11]

Telomere lengths were estimated by a bioinformatic analysis [19] and also by q-PCR. Because telomere length is known to be dependent on methods of DNA extraction and storage [20], this analysis was restricted to the UK families, which were processed and stored in the same laboratory as the identified family. Within the datasets there was evidence of a reduction in telomere length with age and differences by gender; however, because of sample sizes only the qPCR analysis data by age showed a significant age effect (data not shown). The bioinformatic analysis examined whole exome data from the UK multi-case melanoma families. The telomere length of the *TERT* promoter variant carriers was within the range shown for non-mutation carriers (Fig. 2 and Supplementary Fig. 1) (*p* = 0.70 for difference in median adjusted telomere length compared to telomeres from persons without a known germline mutation). The *POT1* mutation carriers showed the longest telomeres as previously reported (*p* < 0.005 compared to persons without germline mutations) [11]. These data were previously published [11] but the figure is updated to indicate that two of the samples have subsequently been shown to have the *TERT* promoter mutation. In the q-PCR analysis, the telomere length of the two c.−57 T>G *TERT* variant carriers (T1 and T2 in Fig. 1) was compared to that of 250 cases without a germline mutation but the assay repeatedly failed quality control for one of the samples (T2). T1 had an age-adjusted telomere length which was at the 10 % percentile of telomere length among those without a germline mutation (*i.e.* had shorter telomeres than the majority of other cases) while once again *POT1* mutation carriers had the longest telomere length (p < 0.001 compared to non carriers, data not shown).

## Discussion

In this report, we describe the contribution of inherited *TERT* promoter mutations to melanoma susceptibility by screening samples from 675 melanoma families consisting of 273 with 3 or more cases and 402 with 2 cases of melanoma. We identified the previously reported c.−57 T>G *TERT* promoter variant in a single 7-case family from the UK, providing further support for this germline mutation being a very rare high penetrance melanoma susceptibility allele. This germline *TERT* promoter variant was identified previously by Horn et al. making this only the second family that has been identified carrying this variant worldwide. From the size of our study and the lack of other families with this mutation in the initial study, it is clear that this inherited mutation is extremely rare in melanoma families. A strength of this report is that we have screened a substantial number of melanoma families from Australia, Denmark, the Netherlands and the UK.

While a detailed assessment of the contribution of this variant to melanoma risk is not feasible, we can put these findings in some context. The UK family with the promoter variant was identified came from a series of 139 three-or-more case melanoma families, of which 56 (40 %) have a *CDKN2A* mutation; 2 (1.4 %) have a *CDK4* mutation, 4 (2.8 %) have shelterin mutations, and now 1 (0.7 %) has a *TERT* promoter mutation.

In the two *TERT* mutation positive families, there are a large number of melanoma cases and other cancers. Both families had mutation carriers with bladder cancer. It is of interest that analyses from The Cancer Genome Atlas (TCGA) identified the *TERT* promoter to be a somatic mutation hotspot for melanoma and bladder cancer as well as glioblastoma, glioma, liver, medulloblastoma and lung cancer [21]. It is speculative but *TERT* could predispose to a broader range of cancers than melanoma.

Small numbers preclude a statistical analysis of the numbers of naevi and atypical naevi in *POT1* and *TERT* mutation carriers but the *POT1* mutation carriers have a number of atypical naevi in the top 10 % of atypical naevi among our cohort of melanoma cases (data not shown). This might be related to factors above and beyond the presence of the *POT1* mutation (*e.g.* behaviour in the sun); a much larger study is required to make more informed comments about the naevus phenotype.

*TERT* encodes the enzyme telomerase reverse transcriptase, a subunit of telomerase, which maintains telomere ends by addition of the repeat TTAGGG. Overexpression of telomerase is key component of the transformation process in many malignant cancer cells. The c.57 T>G mutation is hypothesised to create an ETS transcription factor binding site in the *TERT* promoter and so enhance transcription of the *TERT* gene [1]. The rs2853669 polymorphism (at c.−246) detected in conjunction with c.−57 T>G in both UK and reported samples has also been reported to disrupt an ETS binding site and was associated with low telomerase activity in patients with non small cell lung cancer [22]. The loss of this ETS binding site might be expected to moderate the effect of the ETS site created by the mutation at c.−57, however the combination of both rs2853669 and c.−57 T>G appears to result in a greater increase in promoter activity than the presence of c.−57 T>G alone [1].

Recently, predicted telomere length (based upon inheritance of SNPs identified in genome-wide association studies to be associated with telomere length) was reported to be a risk factor for melanoma [16]. That predicted telomere score has been established as a strong risk factor for melanoma in the general population [16], and germline *POT1*, *TERT* and other shelterin complex gene mutations have been found in melanoma families, suggests that telomere function is critical in melanoma susceptibility. In this study we did not demonstrate increased telomere length in the lymphocyte of *TERT* promoter mutation carriers, in contrast to our recent study in which we did see long telomeres in families with inherited mutations in *POT1* [11]. The implication is that the *TERT* promoter mutation may disrupt the function of the telomere in ways other than by simply increasing its length, as evidenced by findings of common variants that affect cancer risk but not directly via telomere length [20] Alternatively the effect on telomere length may be tissue-specific and the effect differs between melanocytic and lymphocytic.

## Electronic supplementary material

Supplementary material 1 (PDF 144 kb)
