# Supplementary material for: Germline TERT promoter mutations are rare in familial melanoma
Source: Fam Cancer. 2015 Oct 3;15:139–44. doi: 10.1007/s10689-015-9841-9 (PMC4698275; doi:10.1007/s10689-015-9841-9)

Supplementary Figure 1

Q-PCR analysis of telomere length among persons with germline high penetrance mutations in *POT1*, *CDKN2A* and the *TERT* promoter as well as other members of the shelterin complex and the telomere-associated protein *TEP1*. Each data point reflects the deltaCT value (average of at least 2 replicate analyses) for a sample recruited at the defined age in either the Leeds Melanoma Cohort (LMC) [4], a population-based series of melanoma cases, or with the gene name for samples with high penetrance mutations. Also shown is the regression line by age estimated from the LMC cases. Because these results relate to a q-PCR analysis, longer telomeres are identified by more negative DeltaCT values. The only sample with the *TERT* promoter variant for which the assay produced a result has an estimated telomere length less than expected for age at recruitment.

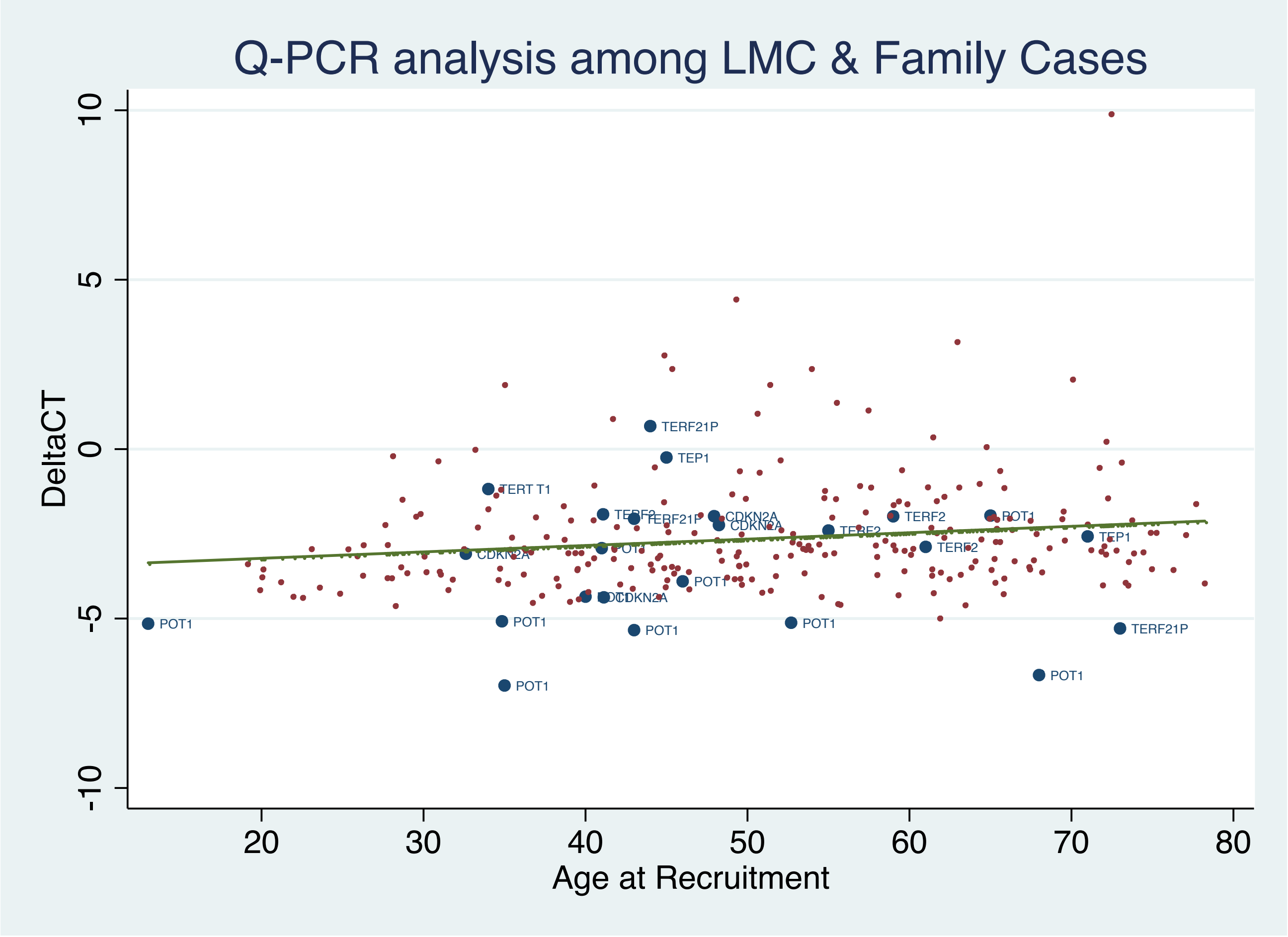

Supplement: Supplementary file 1 — Supplementary material 1 (PDF 144 kb) [file 10689_2015_9841_MOESM1_ESM.pdf]
